# Protocol for an acceptability and feasibility study of sensor‐instrumented SmartSocks® for use by people with dementia

**DOI:** 10.1002/alz.086878

**Published:** 2025-01-09

**Authors:** Byron Creese, Jacqui Arnold, Joanne McDermid, Antonieta Medina‐Lara, Zahinoor Ismail, Zeke Steer

**Affiliations:** ^1^ Brunel University London, London United Kingdom; ^2^ Milbotix Ltd, Oxford United Kingdom; ^3^ The University of Exeter, Exeter United Kingdom; ^4^ University of Exeter, Exeter United Kingdom; ^5^ Hotchkiss Brain Institute, University of Calgary, Calgary, AB Canada; ^6^ Milbotix Ltd, Chipping Norton, Oxfordshire United Kingdom

## Abstract

**Background:**

Currently ∼50% of people with dementia experience behavioural symptoms linked to unmanaged distress. Effective and safe management of these symptoms is critical to maintain the quality of life and overall care of people with dementia. Technological solutions have the potential to help with research into these symptoms. Milbotix are a health tech start‐up who are developing ‘SmartSocks®, a sock‐based wearable that provides wellbeing insights: notably by continually monitoring the physiological state of the wearer to recognise early signs of distress. In this study we will assess the acceptability and feasibility of SmartSocks® for clinical research in dementia in a care home environment.

**Method:**

30 people with dementia living in care homes in the UK will be asked to wear the SmartSocks®. We will conduct a survey of care home staff to assess the acceptability of the SmartSocks® so that we can ensure the socks are suitable for the care home environment. We will then carry out a focus group with care home staff to evaluate the integration of the SmartSocks® into care planning so that we can determine how care homes use the socks alongside best practice in care. We will determine the concurrent validity of SmartSocks® data with established measures of agitation and pain so that we can evaluate the potential of the socks to be used to deliver better care and used as objective outcome measures in clinical trials. Finally, we will estimate the cost of providing the socks and will assess if there are differences between the groups in resource use utilization and health related quality of life data.

**Result:**

We present a protocol for feasibility and acceptability study of a novel wearable technology.

**Conclusion:**

The study will start recruiting in July 2024 and finish in December 2024. Results will be used to inform the design of larger substantive studies that will test the efficacy of SmartSocks® in managing and measuring agitation and distress in care homes.

